# Monthly Summary

**Published:** 1869-06

**Authors:** 


### MONTHLY SUMMARY.

*Death from Hypodermic Injection.*—Lantesson reports that he saw a child die in a few moments with convulsions, after he had injected several drops of liquor ferri sesquichlor. for nævus maternus. Dissection revealed large coagula in the roots of the great veins at the heart and in the right auricle and ventricle. He supposes that a vein of some size was wounded, and that the astringent thus got into the general circulation, coagulated the blood, and finally produced paralysis of the heart. He recommends that the flow of blood into neighbouring venous plexuses should be prevented by pressure when we perform this operation. —*St. Louis Med. Reporter.*

*Danger of Giving Strong Doses of Camphor.*—A case illustrating the above has recently been brought under the notice of the Société de Médicine et de Pharmacie de Grenoble. A enema consisting of five grammes of camphor dissolved in the yolk of an egg, was given to a child three years of age, suffering from typhoid fever. Symptoms of poisoning at once manfested themselves; convulsions, lividity of countenance, stupor, arrest of the urinary secretion, etc. The employment of coffee sufficed to restore the child.—*Ibid.*

*The Three Varieties of Dyspepsia.*—By Dr. Henry Browne M. A., Manchester. He recognizes three principal kinds of Dyspepsia, which he calls respectively sulphuretted hydrogen dyspepsia, or that accompanied with "rotten egg" eructations; carbonic acid dyspepsia or that evidenced by tasteless eructations; and butyric acid dyspepsia in which the eructations are sour or acrid. To a patient suffering from the first named of these he recommends abstinence from meat and eggs, and prescribes farinaceous diet, along with a mixture containing strong hydrochloric acid, chlorate of potash, filled up with a vegetable bitter. In the case of patients afflicted with carbonic acid dyspepsia he orders a lean meat diet and an avoidance of bread, potatoes, and farinaceous diet generally; while to the third class he prescribes the use of sugar and fat. Dr. Browne is fond of pointing out the incalculable harm done to the digestive system by an immoderate indulgence in tea, and in his, as in every medical out-patient room, he finds abundant illustrations of his observations. These cases he styles "tea dyspepsia," and he relies for a cure of them upon a daily allowance of wine, regularity of meals, and abstinence from tea. He insists very strongly on the necessity of wearing flannel, especially in the rheumatic diathesis, enjoining upon his patients the use not only of flannel jackets or shirts, but also of flannel drawers and woolen stockings; and he invariably adds this precept—"Never wear during the night the flannel clothing you have worn during the day, but change your flannel garments night and morning, taking care to have them well aired and dried in the meantime.—*Medical Times and Gazette,and Braithwaite.*

---

*Excessive Sweating of the Hands or Feet.*—For the relief of this troublesome affection, Dr. Donilt recommends the thorough application of the hottest water that can be borne without pain to the offending parts until they are red hot and tingling as if scalded. This treatment the author states, sometimes appears to aggravate the affection. Hebra recommends the frequent local use of a solution containing one drachm of tannic acid mixed in six ounces of alcohol; this liquid should be rubbed into the parts several times a day, and the skin must not afterwards be wiped; a little powdered asbestos is to be sprinkled on it while still wet and with this the part is to be rubbed till it is dry.—*American Eclectic Medical Review.*

*Sleeping Sickness* (*Maladie du Sommeil,*)—A short article with the above title, by Dumoutier, a surgeon in the French Navy, appears in the *Gazette des Hospitaux*, Oct. 13, 1868. The disease was met with on the coast of Africa, and especially in the territory of Gabo and Congo. There is an irresistible tendency to sleep, accompanied with no suffering, but with a general weakening of the limbs ; the gait is uncertain, and the tactile sensibility seems perverted. During the sleep, the fæces and urine are voided involuntarily. Intelligence seems unaffected, respiration is normal and digestion regular. The patients are shunned by their companions.

The disease is observed more especially among the slaves or captives from the interior, who have undergone great suffering and performed excessive work with insufficient and bad food—and who are the victims of chagrin, ennui and despair.

Strychina, tonics, exercise and electricity were employed, all to no purpose.

At two autopsies, no change was found either in the brain, the cord, or their membranes.—*Boston Medical & Surg. Jour.*

---

*The Bi-sulphide of Carbon.*—We have several times called attention to this curious substance. Recently in an English journal Dr. G. Kennion speaks of it as a cure for headache. He says : Its mode of application is simple. A small quantity of the solution (about two drachms) is poured upon cotton wool, with which a small, wide-mouthed, glass stoppered bottle is half filled. This, of course, absorbs the fluid, and when the remedy has to be used, the mouth of the bottle is to be applied closely (so that none of the volatile vapor may escape) to the temple, or behind the ear, or as near as possible to the seat of pain, and so held for from three to five or six minutes. After it has been applied for a minute or two a sensation is felt as if several leeches were biting the part; and after the lapse of two, three or four minutes more, the smarting and pain become rather severe, but subside almost immediately after the removal of the bottle. It is very seldom that any redness of the skin is produced. The effect of this application, as I have said, is generally immediate. It may be reapplied, if neccessary, three or four times in the day.

The class of headaches in which this remedy is chiefly useful is that which may be grouped under the wide term of "nervous." Thus neuralgic headache, periodic headache, hysterical headache, and even many kinds of dyspeptic headache, are relieved by it; and although the relief of a symptom is a very different affair, of course, from the removal of its cause, yet one who has witnessed (and who of us has not seen?) the agony and distress occasioned by severe and repeated headache, but must rejoice in having the power of affording relief in so prompt and simple a manner.

As regards the *modus operandi* of this remedy, it is difficult, perhaps, to form a certain opinion; but I am disposed to attribute it to the sedative effect of the vapor of the bisulphide, absorbed through the skin, and acting upon the superficial nerves of the part to which it is applied. The remarks of M. DELPECH (*Annales d'Hygiene*, January 1863,) point out very clearly the remarkable prostration of the whole nervous system produced in workmen who, in certain manufactures, are exposed to the vapor arising from a solution of the bisulphide of carbon; and we can readily understand that a somewhat similar effect, upon a small scale, may be produced by the application of this vapor to a limited portion of the surface.—*Med. & Surg. Reporter.*

